# Muscle Fatigue Assessment in Healthcare Application by Using Surface Electromyography: A Transfer Learning Approach

**DOI:** 10.3390/s26020654

**Published:** 2026-01-18

**Authors:** Andrea Manni, Gabriele Rescio, Andrea Caroppo, Alessandro Leone

**Affiliations:** Institute for Microelectronics and Microsystems, National Research Council of Italy, 73100 Lecce, Italy; andrea.manni@cnr.it (A.M.); andrea.caroppo@cnr.it (A.C.)

**Keywords:** muscle fatigue, surface electromyography, transfer learning

## Abstract

Monitoring muscle fatigue is essential to ensure safety and support activity in populations such as the elderly. This study introduces a novel deep learning framework for classifying muscle fatigue levels using data from wireless surface electromyographic sensors, with the long-term goal of supporting applications in Ambient Assisted Living. A new dataset was collected from healthy elderly and non-elderly adults performing dynamic tasks under controlled conditions, with muscle fatigue levels labelled through self-assessment. The proposed method employs a pipeline that transforms one-dimensional electromyographic signals into two-dimensional time–frequency images (scalograms) using the Continuous Wavelet Transform, which are then classified by a fine-tuned, pre-trained Convolutional Neural Network. These images are then classified by pretrained Convolutional Neural Networks on large-scale image datasets. The classification pipeline includes an initial binary discrimination between non-fatigued and fatigued conditions, followed by a refined three-level classification into No Fatigue, Moderate Fatigue, and Hard Fatigue. The system achieved an accuracy of 98.6% in the binary task and 95.6% in the multiclass setting. This integrated transfer learning pipeline outperformed traditional Machine Learning methods based on manually extracted features, which reached a maximum of 92% accuracy. These findings highlight the robustness and generalizability of the proposed approach, supporting its potential as a real-time, non-invasive muscle fatigue monitoring solution tailored to Ambient Assisted Living scenarios.

## 1. Introduction

Muscle fatigue is a neuromuscular phenomenon characterized by a decrease in the muscle’s ability to generate force, typically experienced as a subjective sensation of tiredness or an objective decrease in performance [1,2]. Resulting from prolonged or repetitive muscle activity, it is associated with physiological changes such as reduced muscle fibre conduction velocity, altered motor unit recruitment patterns—often involving greater reliance on larger, higher-threshold motor units (type II fibres)—and changes in motor unit firing rates [3].

This condition is particularly relevant for elderly people, often dealing with age-related declines such as sarcopenia, decreased muscular endurance, and slower recovery rates [4]. Early and objective fatigue detection in this population is essential to support adaptive physical activity, prevent overexertion, and improve rehabilitation outcomes [5]. Muscle fatigue monitoring is a crucial physiological parameter in the Ambient Assisted Living (AAL) domain, providing valuable information on the health status, activity level, and immediate risk of an elderly with chronic diseases [6,7]. For example, it can be used in the context of home rehabilitation after surgery or a stroke event where the system can guide the user, indicating when a muscle is too fatigued and they should stop exercising to avoid injury, or when they are not working hard enough. Another context could be the management of chronic and neurodegenerative diseases such as Multiple Sclerosis, Parkinson’s Disease, Sarcopenia, and Heart Failure. In this context, an AAL system, for example, could adapt the environment according to the perceived muscle fatigue level (e.g., suggesting to turn on the air conditioning if the temperature affects fatigue, as in Multiple Sclerosis), which is a common symptom. In summary, muscle fatigue monitoring in AAL transcends the simple control of a physiological parameter. It becomes a proactive tool to prevent adverse events, personalise care, maintain autonomy, and significantly improve the quality of life of frail people.

Muscle fatigue can be assessed using both invasive and non-invasive methods. While invasive techniques such as muscle biopsy and needle electromyography (EMG) provide detailed physiological data, they are impractical for routine or home-based use. Non-invasive alternatives include endurance tests, imaging, and surface electromyography (sEMG) [8]. In particular, sEMG is currently becoming a widely adopted tool due to its wearability, non-invasiveness, and ability to record the electrical activity of muscle contractions [9].

Conventional self-assessment tools, e.g., the Borg RPE scale [10], and performance-based assessments are still popular, although limited by subjectivity, dependence on motivation, and poor suitability for real-time and unsupervised monitoring. In contrast, sEMG enables objective, continuous monitoring and is responsive to both voluntary and reflexive muscle activity. Nonetheless, sEMG signals are also susceptible to noise and inter-subject variability and require expert interpretation.

To address these challenges, several studies have applied Machine Learning (ML) to classify fatigue states from sEMG features. Time-domain and time–frequency features have proven effective, particularly when combined with robust classifiers. For instance, Karthick et al. [11] employed high-resolution time–frequency techniques—such as the Stockwell Transform, B-distribution, and Extended Modified B-distribution (EMBD)—along with feature selection via Genetic Algorithms and Binary Particle Swarm Optimization, achieving a 91% classification accuracy using EMBD features and a polynomial Support Vector Machine (SVM).

In addition to simple fatigue detection, more recent works have focused on tracking fatigue progression. Punitha et al. [12] proposed the use of geometric features from the complex-plane representation of sEMG (via Hilbert Transform), combined with XGBoost and SHAP explainability methods, achieving an F1-score of over 95% in fatigue phase classification. Similarly, Wang et al. [13] integrated sEMG with wearable textile sensors for measuring muscle thickness. Their SVM-based model demonstrated high predictive performance, with an F1-score of 95% and an AUC of 98.7%.

Pang and Tang [14] proposed a method for muscle fatigue classification based on sEMG signals, combining Principal Component Analysis (PCA) with a Random Forest (RF) algorithm. Tested on eight subjects, the model achieved an accuracy of 93.3%, demonstrating the effectiveness of the combined use of dimensional reduction and feature selection. However, many existing approaches still rely on manually engineered features and limited, often homogeneous cohorts of subjects, potentially reducing their applicability to real-world monitoring systems for elderly. Recent efforts in Deep Learning (DL) have recently started to overcome these problems, using architectures capable of automatically extracting features from raw or minimally processed sEMG data. Wu et al. [15] proposed a hybrid architecture combining Temporal Convolutional Networks (TCNs), self-attention mechanisms, and Convolutional Neural Networks (CNNs), reaching 90.07% accuracy in fatigue classification during isometric contractions. However, DL methods generally require large annotated datasets, often difficult to obtain in physiological studies. However, challenges remain in constructing models with good generalisation across individuals, especially in scenarios involving dynamic tasks and natural fatigue progression. Transfer learning (TL) provides a promising solution, allowing pre-trained models to be adapted for fatigue classification tasks, even with limited sEMG data [16].

This work presents a methodological framework validated on a cohort of healthy adults. Its performance on this cohort establishes a proof-of-concept and technical benchmark, which is a necessary first step before future clinical validation with target patient populations. The framework uses sEMG signals collected from upper limb muscles during dynamic exercises for the automatic classification of muscle fatigue levels. To convert 1D sEMG data into 2D image-like representations suitable for CNN, a DL architecture based on TL and Continuous Wavelet Transform (CWT)-based scalograms is employed [17]. While DL offers the potential for automatic feature extraction, its application in physiological monitoring is often hampered by two interconnected challenges: the high cost and difficulty of acquiring large, annotated sEMG datasets and the significant inter-subject variability in sEMG signals due to anatomical and physiological differences. Training a robust DL model from scratch typically requires a volume of data that is impractical for many clinical or research settings, and models trained on small, subject-specific datasets often fail to generalize to new individuals. TL provides a powerful framework to directly mitigate these issues. By leveraging features learned from very large-scale image datasets (e.g., ImageNet), a pre-trained Convolutional Neural Network (CNN) already possesses a rich hierarchy of generic feature detectors (e.g., for edges, textures, and patterns). Because it is pre-trained on a vast and diverse dataset, this foundational model has a strong regularization effect, making it inherently less susceptible to overfitting when fine-tuned on small datasets. When this model is then fine-tuned on our sEMG scalograms, it efficiently adapts these general-purpose features to the specific domain of time–frequency representations of muscle activity. This process allows the network to achieve high performance with a limited amount of data. Furthermore, by training on a diverse set of subjects, the TL model learns a more generalized representation of fatigue that is inherently more robust to inter-subject variability compared to a model trained from scratch on a small cohort, as it is regularized by its vast prior knowledge.

Fatigue is annotated using both self-referenced effort levels and temporal segmentation, allowing classification into multiple fatigue states. The main contributions of this study are as follows:

- the acquisition of a dedicated sEMG dataset from both healthy elderly and non-elderly people performing repetitive upper limb movements under controlled conditions;
- a TL-based fatigue classification pipeline applied to time–frequency representations of sEMG signals;
- An experimental evaluation demonstrating that the proposed approach achieves higher accuracy than traditional ML classifiers based on features, with promising potential for integration into real-time monitoring systems in AA contexts.

The remainder of this paper is organized as follows: Section 2 describes the materials and methods, including the data acquisition protocol and classification pipeline. Section 3 presents the experimental results comparing them with reference approaches. Conclusions and future research are presented in Section 4.

## 2. Materials and Methods

**Figure 1:**
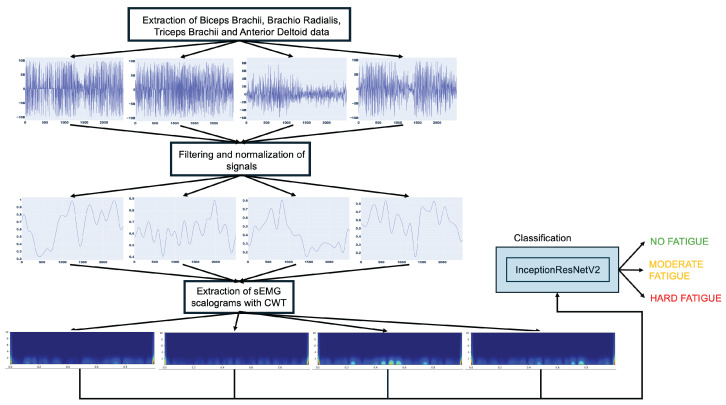
Proposed muscle fatigue detection methodology. The CWT scalograms visualize the time–frequency content of the sEMG signal, where color intensity represents the magnitude of the signal’s energy at a given time and frequency.

In this paper, an intelligent sEMG scalogram scheme for detecting muscle fatigue level is proposed. In the presented approach, four upper limb muscles’ data are extracted from the sEMG signal obtained from a purpose-built dataset. From the study of the literature, the Biceps Brachii (BB), Brachio Radialis (BRA), Triceps Brachii (TRI), and Anterior Deltoid (DEL) were identified and the data extracted from these muscles are filtered and segmented into fragments of 2500 samples (one sample per millisecond) that are transformed into images using CWT. The resulting scalograms are sent to the deep neural network for accurate classification between no fatigue and fatigue in a first scenario and No Fatigue, Moderate Fatigue, Hard Fatigue in a second scenario. Figure 1 presents a schematic diagram of the proposed methodology.

### 2.1. System Description

The developed sEMG platform integrates a wearable hardware component for signal acquisition and a customized software interface for real-time visualization and data storage. This modular setup supports user-friendly operation and facilitates protocol-based data collection. The hardware relies on the FREEEMG1000 system (BTS Bioengineering, Garbagnate Milanese, Italy) [18]. The system employs lightweight (13 g), wireless sEMG probes with dimensions of 41.5 mm × 24.8 mm × 14 mm. Up to ten probes can operate simultaneously, each connected to pre-gelled Silver/Silver Chloride (Ag/AgCl) electrodes. These active electrodes amplify and digitize the signals on-board and transmit them wirelessly to a USB receiver connected to a tablet. Thanks to the wireless configuration and integrated rechargeable batteries, the probes support up to 6 h of continuous streaming, enabling full freedom of movement during dynamic tasks.

A custom software tool was developed to visualize raw sEMG waveforms in real-time and store data for offline processing. Acquisition protocols can be uploaded into the system and customized for each session.

In this study, BB, TRI, BRA, and DEL were selected because they represent the main agonist, antagonist, and synergist muscles involved in elbow flexion during the bicep curl exercise. Previous works have demonstrated the relevance of these muscle groups in this task: Marcolin et al. [19] compared the electromyographic activity of BB and BRA across different curl variants, while Coratella et al. [20] reported distinct excitation of BB and BRA depending on the handgrip type, highlighting the role of synergistic activation. Other studies have investigated fatigue monitoring using EMG but restricted the analysis to BB only, explicitly recognizing the exclusion of other synergist muscles as a limitation [12,13,21]. Finally, Aghamohammadi-Sereshki et al. [22] included BB, BRA, and TRI in their fatigue analysis using EMG features; although this work was presented as a conference paper, it further supports the relevance of these muscles for assessing fatigue during elbow flexion tasks. DEL was also considered, since the cohort consisted of non-expert participants. In such conditions, the exercise may not always be performed with perfect technique, potentially involving compensatory activation of synergist or stabilizing muscles. Monitoring this muscle therefore ensures a more comprehensive assessment of fatigue during the bicep curl task.

**Figure 2:**
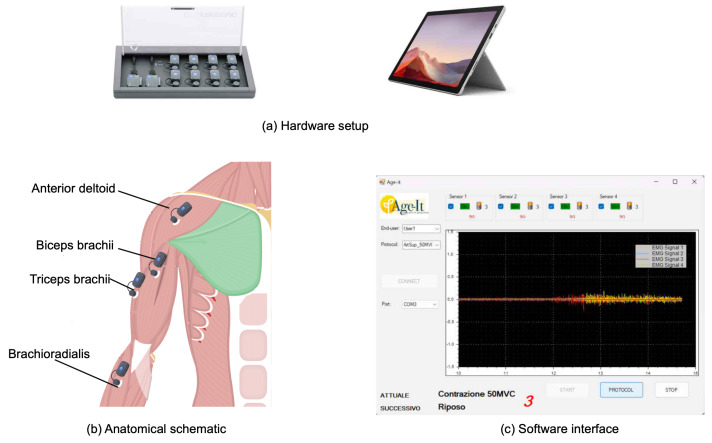
Overview of the sEMG acquisition platform. (**a**) Hardware setup: BTS FreeEMG 1000 wireless sEMG system with probes and tablet. (**b**) Anatomical schematic indicating the four monitored upper-limb muscles: Biceps Brachii, Brachioradialis, Triceps Brachii, and Anterior Deltoid. (**c**) Custom software interface for real-time sEMG visualization and protocol configuration during data acquisition.

Electrodes were placed along the muscle fiber direction with an inter-electrode distance of approximately 20 mm to maximize the amplitude of the sEMG signals. In Figure 2 an overview of the sEMG platform is provided.

### 2.2. Data Acquisition

A total of 20 healthy adults (average age = 54.5 ± 22.2 years; range: 29–78 years) were recruited. sEMG signals with a sampling frequency of 1000 Hz were collected during the execution of repeated bicep curls using a 2 kg dumbbell. The bicep curl was chosen because it offers a simple, safe, reproducible, and highly measurable model to study how a single muscle group loses force over time. Furthermore, its biomechanical simplicity allows researchers and clinicians to accurately isolate and quantify the physiological changes associated with fatigue, making it a gold standard for many testing protocols. Before the fatigue protocol, the Maximum Voluntary Contraction (MVC) of the BB muscle was recorded via isometric elbow flexion against a fixed resistance. Participants, seated with their elbow at 90°, performed three 5 s maximal contractions with 10 s rest intervals. The mean of the values thus acquired was used to normalize the processed data. During the fatigue protocol, participants performed continuous bicep curls at a controlled pace. To define the level of fatigue in the patients under examination and label the acquired electromyographic signals accordingly, the following criteria were applied: every three repetitions they self-reported their perceived fatigue on a 0–10 numerical rating scale, according to the Borg Scale [23]. The session was interrupted when the participant was unable to complete a full curl. On average, each recording session lasted approximately 4–5 min for the elderly participants. Each trial was temporally segmented into three intervals (early, middle, late task stages). These intervals were matched with self-reported fatigue scores and categorized into one of three fatigue classes, as shown in Table 1. In cases of disagreement between the temporal phase and the self-reported Borg score, the higher fatigue class was conservatively assigned. This decision was guided by a safety-first principle for the intended AAL application, where failing to detect fatigue (a false negative) poses a higher risk than a false positive alert. However, this labeling strategy introduces a deliberate, systematic bias toward the positive class (fatigue). As a result, the model trained on these labels is optimized for high sensitivity in a precautionary monitoring context rather than for a strictly neutral, physiology-only classification. This constitutes a recognized methodological limitation of the present study. Consequently, the reported classification performance metrics—particularly recall and accuracy—should be interpreted with an understanding that they reflect the model’s ability to replicate a safety-biased ground truth, not an objective physiological state. Future work incorporating instrument-based fatigue biomarkers is necessary to establish an unbiased ground truth for purely physiological validation. The most common scenario was a participant reporting a Borg score in the “Moderate Fatigue” range (3–5) during the “early” temporal phase (0–33% of task progress). In these cases, the segment was labeled “Moderate Fatigue” rather than “No Fatigue”. This method may introduce a bias towards the positive class (fatigue). However, we believe that for this specific healthcare application, this bias is deliberate and justifiable. A model trained in this way will be more sensitive to early signs of fatigue, which is clinically preferable. Furthermore, the high performances achieved in multi-class classification and presented in Section 3, particularly the high F1-scores, suggest that the model has successfully learned the underlying patterns associated with each class despite this labelling strategy.

**Figure 3:**
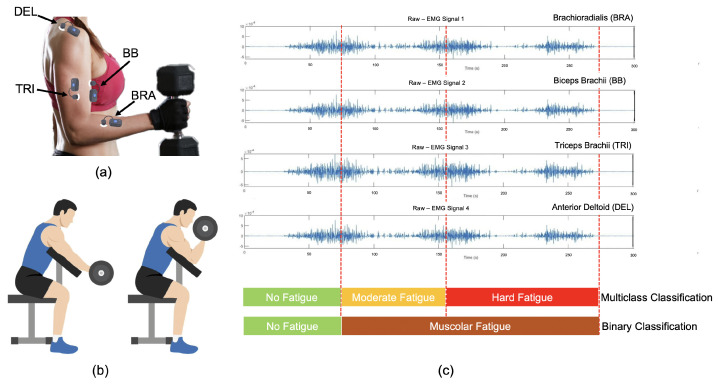
Experimental protocol: (**a**) probes positioning; (**b**) executed task: bicep curls until task failure; (**c**) signal segmentation and labeling according to both temporal phase and perceived fatigue.

The labeling process was performed collaboratively by the participant and the operator, who supervised the execution and annotated muscle fatigue levels in real-time. In Figure 3 the probes’ positioning, the executed tasks, and the signal labeling are reported.

It is important to emphasize that the defined fatigue classes (No, Moderate, Hard) are based on a compound criterion integrating time-on-task and subjective perception, not on a direct, objective physiological measure such as a decline in force output or a specific shift in the sEMG power spectrum. While this pragmatic approach generates labels suitable for training a pattern-recognition model in a safety-oriented context, it means the model learns to associate sEMG patterns with this compound state rather than with isolated physiological fatigue. Therefore, the system is more accurately described as a monitoring of “perceived exertion and performance deterioration” during a repetitive task, which is a highly relevant parameter for AAL safety, but not a direct measure of localized muscle fatigue as defined physiologically (e.g., by a drop in median frequency).

### 2.3. Pre-Processing

In this phase, the following steps are performed: (a) noise reduction, (b) EMG enveloping, and (c) data normalization. In step (a), baseline noise and signal artefacts due to EMG electrode movements [24] are reduced with a 4th-order Butterworth bandpass filter with a frequency between 20 Hz and 450 Hz.

Next, in (b), to achieve comparable signals suitable for further processing, the linear envelope of the signal was determined using full rectification and a Butterworth low-pass filter with a cutoff frequency of 10 Hz [25]. Lastly, in (c), the normalization step is performed as described in Section 2.2.

### 2.4. Feature Extraction

In the feature extraction phase, pertinent information is extracted from the sEMG signal concerning fatigue or muscle fatigue levels. In this study, several time-domain and frequency-domain features used for monitoring upper limb muscles were analysed [26,27,28]. This phase is necessary for the ML classifiers to compare and validate the proposed TL approach. The chosen time-domain and frequency-domain features are well-established in the sEMG literature for fatigue analysis, as they directly reflect the known physiological changes that occur in fatiguing muscle. Specifically, the studied features are as follows:

- Time-Domain Features: These are related to the amplitude of the sEMG signal. During a fatiguing contraction, the body recruits additional motor units and decreases their firing rates to maintain force. This often leads to an increase in signal amplitude, which is captured by Root Mean Square (RMS) and Mean Absolute Value (MAV). The Waveform Length (WL), which represents the cumulative length of the signal waveform, also tends to increase with this amplitude change and signal complexity.
- Frequency-Domain Features: These features characterize the spectral content of the signal. Muscle fatigue is physiologically linked to a decrease in muscle fiber conduction velocity. This slower conduction velocity causes a compression of the power spectrum towards lower frequencies, manifesting as a decrease in the Mean and Median Power Frequencies (Mean Frequency (MNF), Mean Power Frequency (MPF), Median Frequency (MDF)). This spectral shift is one of the most robust indicators of localized muscle fatigue.
- Complexity and Spike-Based Features: Features like Zero Crossing (ZC) and Willison Amplitude (WAMP) provide information on the signal’s frequency content and spike patterns in the time domain. The changes in motor unit firing patterns and the shape of the action potentials during fatigue can alter the signal’s complexity and the incidence of rapid fluctuations, which these features help to quantify.

Table 2 shows the analyzed features.

The sliding window size was fixed at 2500 ms, with an incremental window of one-tenth of the sliding window. After segmenting the EMG data, eight EMG features were extracted from each of the four channels, resulting in a feature size vector of 32.

To prevent data leakage and ensure a rigorous evaluation of cross-subject generalizability, the Leave-One-Subject-Out (LOSO) cross-validation split was performed prior to the windowing procedure. For each fold, all raw sEMG data from the designated test subject were held out. The sliding window segmentation (2500 ms window, 250 ms increment) was then applied independently to the continuous data of the training subjects and to the held-out test subject’s data. This guarantees that no segment from the test subject, nor any overlapping or derived window from it, is present in the training set. The same strict subject-level separation was applied during the generation of CWT scalograms for the TL pipeline.

### 2.5. Continuous Wavelet Transform

The TL application to the muscle fatigue detection is expected to use data from other users to generate general features and improve classification performance with a pre-trained CNN of a new user, since less data are needed to set up the training.

These pre-trained networks require 2D images as input, whereas the sEMG signal is 1D. For this purpose, a time–frequency representation (TFR) [29] of the sEMG signal was evaluated, since the resulting images contain the variation of amplitude and frequency over time. To extract the TFR of the sEMG signals, CWT was employed, which, by decomposing a signal into a set of “wavelets”, is able to detect the time–frequency information [30]. A two-dimensional image, known as a scalogram, in which the *x*-axis is time and the *y*-axis is frequency, is thus obtained, identifying signal features not visible in the time or frequency domains alone. Given a signal s(t), the corresponding CWT is given by the following expression:

(1) $${\mathrm{CWT}}_{s(a,b)}=\int s\left(t\right)\varphi_{a,b}^{*}\left(t\right)dt$$

where *a* and *b* are the scale and time value, respectively (a>0, a,b∈R), and $\varphi_{a,b}^{*}\left(t\right)$ is the analyzed mother wavelet.

In the proposed approach, CWT was obtained with the Morlet wavelet as the mother wavelet, setting the scale value to 256 and obtaining a scalogram representing the absolute value of the CWT coefficients. The Morlet wavelet was selected as the mother wavelet for the CWT due to its excellent balance between time and frequency localization. Furthermore, its sinusoidal nature under a Gaussian envelope provides a shape that closely resembles the motor unit action potentials that constitute the sEMG signal. This morphological similarity makes it particularly well-suited for analyzing non-stationary physiological signals like sEMG, as it can effectively capture the time–frequency characteristics of the underlying neuromuscular activity during dynamic fatigue progression.

The scalograms were extracted from the time series of the sEMG signals for BB, BRA, TRI, and DEL muscles, partitioned into 2.5 s time windows. The corresponding scalograms were resized to 224 × 224 to fit the input layer of the selected pre-trained architecture described in Section 2.6.

**Figure 4:**
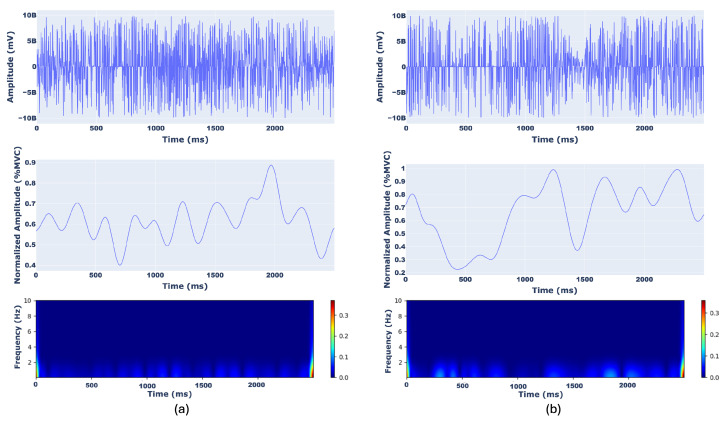
Representation of (top) raw sEMG signal, (middle) filtered and normalized sEMG signal (amplitude expressed as a percentage of Maximum Voluntary Contraction, %MVC), and (bottom) corresponding CWT scalogram for the BB (**a**) and BRA (**b**) muscles. The time axis spans a 2500 milliseconds window. The scalogram color intensity represents the magnitude of the CWT coefficients across frequencies.

**Figure 5:**
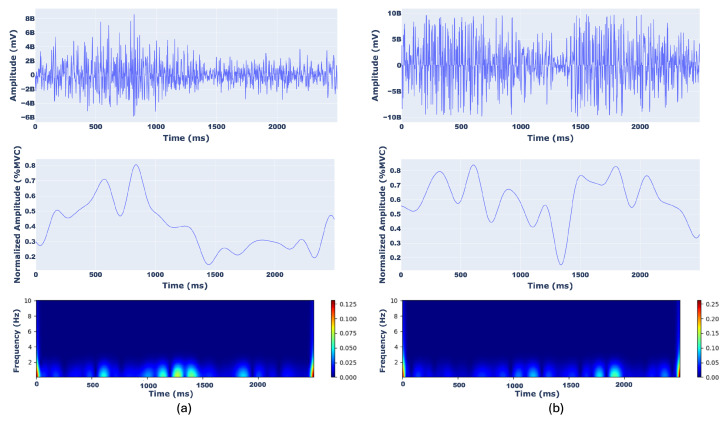
Representation of (top) raw sEMG signal, (middle) filtered and normalized sEMG signal (%MVC), and (bottom) corresponding CWT scalogram for the TRI (**a**) and DEL (**b**) muscles. Axes and colorbar follow the same conventions as in Figure 4.

An example of scalograms from BB (a), BRA (b), TRI (c), and DEL (d) muscles with the corresponding raw and MVC-normalized filtered sEMG signals are represented in Figure 4 and Figure 5, where amplitudes are expressed in %MVC and time in milliseconds.

### 2.6. Classification Approach for Muscle Fatigue

A TL architecture was used in this work to automatically classify the sEMG scalograms into fatigue and no fatigue in one case and into No Fatigue, Moderate Fatigue, and Hard Fatigue in a second case, to limit the main problem of CNN architectures, i.e., the need for large training data, which is particularly important in complex signals such as sEMG. The main motivation for using TL in an sEMG signal application context is the difficulty of determining the most appropriate features for correct classification using classical ML approaches, due to the high complexity of the signal.

In this study, four pre-trained models were used to classify muscle fatigue, in particular InceptionResNetV2, DenseNet121, ResNet50, and InceptionV3. These models were selected based on being some of the most frequently employed in the analysis of sEMG signals and achieving the best performance, although in different contexts [31] to the one presented in this work.

InceptionResNetV2 [32] is an improved version of the InceptionV3 model [33] having better computing power, network depth, and network nonlinearity. Feature extraction is composed of three similar modules, Inception-ResNet-A, Inception-ResNet-B, and Inception-ResNet-C. In particular, the second and third contain asymmetric convolution kernels versus the symmetric convolution kernels of the first.

DenseNet121 [34] is a Convolutional Neural Network architecture distinguished by its dense connectivity pattern. Its core principle is to maximize the flow of information throughout the network by connecting each layer to every other layer that precedes it in the hierarchy. This design ensures that all subsequent layers have direct access to the feature maps from all earlier layers, promoting feature reuse and enhancing efficiency. Structurally, the model is composed of multiple “Dense Blocks”, where layers are densely interconnected, interspersed with “Transition Layers” that control feature map sizes. The complete architecture begins with an input and convolutional layer, followed by a sequence of these dense and transition blocks (which include combinations of convolution and pooling layers), and culminates in a linear output layer for classification.

The ResNet50 [35] architecture, built with 48 convolutional layers and two pooling layers (max and average), is renowned for its residual connections. These skip connections facilitate the learning of residual functions by letting a layer’s input bypass it via an identity map. This design effectively allows the network to skip redundant layers, simplifying the learning process. A significant benefit of this approach is its regularization effect, which helps prevent the model from overfitting to its training set.

The InceptionV3 [33] model improves upon its predecessor, InceptionV1, through the factorization of convolutional layers for parameter optimization. Its structure comprises three core blocks: (1) a basic convolutional block that alternates between convolution and max-pooling for feature extraction, (2) a sophisticated Inception block, and (3) a classifier. To minimize computational overhead, the model employs factorized convolutions, breaking down complex filters into simpler, sequential operations. Consequently, InceptionV3 delivers state-of-the-art accuracy in object recognition, solidifying its status as a widely adopted model for TL.

In this study, a complete fine-tuning strategy was adopted, where all layers of the pre-trained convolutional base were set as trainable. This was chosen to enable maximal domain adaptation from natural images to the time–frequency textures of sEMG scalograms. We acknowledge that with a cohort of 20 subjects, this approach carries a risk of subject-specific overfitting, as the number of model parameters significantly exceeds the number of independent biological samples. To mitigate this risk, we employed two key strategies: (1) LOSO cross-validation, which ensures that the model is evaluated on a completely unseen subject in every fold, providing a stringent test of generalizability across individuals, and (2) the use of strong regularization techniques including dropout layers and a small learning rate (see Table 3).

**Figure 6:**
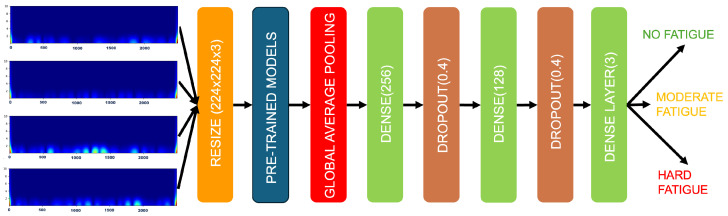
Detailed architecture of the proposed fine-tuned InceptionResNetV2 model for muscle fatigue classification. The input is a 224 × 224 × 3 CWT scalogram. The pre-trained InceptionResNetV2 convolutional base is followed by a Global Average Pooling (GAP) layer. The custom classification head consists of two dense layers (with 256 and 128 units, respectively), each followed by a dropout layer (rate = 0.4), and a final dense layer with softmax activation (2 or 3 units). Key feature map dimensions are annotated. Other training hyperparameters (learning rate, batch size, etc.) are listed in Table 3.

To classify muscle fatigue as accurately as possible, Global Average Pooling was added after the pre-trained architecture to increase the feature importance connection with the class label [36]. Next, a dense layer, a dropout layer, another dense layer, a dropout layer, and a final dense layer with a “softmax” activation feature were appended. The optimization algorithm used is “Adam”, with accuracy as the metric. The developed architecture is depicted in Figure 6, while various network parameters are listed in Table 3. For reproducibility, the architecture specifics are as follows: the input scalogram dimension is 224 × 224 × 3. After the pre-trained base and Global Average Pooling, the first dense layer contains 256 neurons, followed by a dropout layer with a rate of 0.4. A second dense layer of 128 neurons is then applied, followed by another dropout layer (rate = 0.4). The final output layer is a dense layer with a number of units corresponding to the classification task (2 or 3) and a softmax activation function.

Furthermore, five classical ML classifiers were used to validate the proposed approach. In particular, Logistic Regression (LR) [37], RF [38], Decision Tree (DT) [39], SVM [40], and K-Nearest Neighbor (KNN) [39] were evaluated. Table 4 and Table 5 show the selected optimal parameters in each ML model for two and three classes, respectively, resulting from a grid search technique [41].

## 3. Results and Discussion

The model was evaluated using a stringent LOSO cross-validation protocol. Crucially, the subject split was enforced before any windowing or signal transformation, ensuring that the training and test sets in each fold contained data from entirely separate individuals, with no possibility of data leakage through overlapping segments.

Several experiments were executed to evaluate the effectiveness of the proposed approach. The algorithms were implemented using Python 3.8, employing the following main libraries: Tensorflow (2.10), pandas (2.0.3), scikit-learn (1.2.1), and spkit (0.0.9.6.7). The hardware was a Dell^™^ Precision 7920 Rack workstation with 256 GB RAM, two Intel Xeon Gold 5218R CPU@2.10 Ghz processors, and three 12GB NVIDIA^™^ RTX A2000 GPUs.

The following four metrics were considered to analyse the performance of the proposed approach: accuracy (Acc), precision (Pr), recall (Re), and F1-score, specified by the following equations [42]:

(2) $$\begin{array}{ccc} \mathrm{Acc} & = & \frac{\sum_{i=1}^{C}\left({\mathrm{TP}}_{i}+{\mathrm{TN}}_{i}\right)}{\sum_{i=1}^{C}\left({\mathrm{TP}}_{i}+{\mathrm{TN}}_{i}+{\mathrm{FP}}_{i}+{\mathrm{FN}}_{i}\right)} \end{array}$$

(3) $$\begin{array}{ccc} {\mathrm{Pr}}_{i} & = & \frac{{\mathrm{TP}}_{i}}{{\mathrm{TP}}_{i}+{\mathrm{FP}}_{i}} \end{array}$$

(4) Rei=TPiTPi+FNi

(5) $$\begin{array}{ccc} {F1\text{-}\mathrm{Score}}_{i} & = & \frac{2*{\mathrm{TP}}_{i}}{2*{\mathrm{TP}}_{i}+{\mathrm{FP}}_{i}+{\mathrm{FN}}_{i}} \end{array}$$

where TP (True Positive) denotes that the algorithm correctly predicted a muscle fatigue phase; FP (False Positive) denotes that the algorithm identified a fatigue phase that did not occur; TN (True Negative) denotes that the algorithm correctly detected the absence of a fatigue phase; and FN (False negative) implies that the algorithm did not predict a fatigue phase that occurred. Accuracy indicates the ratio of all correctly classified samples to all samples; accuracy in predicting only positive occurrences is shown by precision; the performance of the model in predicting positive occurrences using all positive cases is obtained by recall; the F1-score influences true positive cases more than precision.

Finally, in order to verify the generalisability of the proposed model, performance was verified using a LOSO cross-validation. According to this protocol, one subject was selected for testing and the remaining subjects were used as the training set. Specifically, the LOSO cross-validation tests were replicated for each of the twenty subjects designated as the test set in our evaluation. The high and consistent performance across all LOSO folds (low standard deviation in Table 6 and Table 7) suggests that overfitting to specific subjects was controlled in this instance, but the limited sample size remains a constraint on the model’s proven generalizability to broader populations.

Table 6 shows the obtained results with two classes (No Fatigue and Fatigue) for the considered TL architectures and the comparison with the achieved results using the evaluated ML classifiers. Similarly, Table 7 reports the results with three classes (No Fatigue, Moderate Fatigue, and Hard Fatigue).

From the two tables, it can be seen that the modified InceptionResNetV2 architecture performed best in terms of average accuracy compared to the ML classifiers. In fact, after a training period of 70 epochs, it achieved an accuracy of 98.62% with two classes, which is about 6.6% higher than the RF that performed best among the ML classifiers. Considering three classes, the average accuracy achieved by InceptionResNetV2 is 95.64%, about 9% higher than the best ML classifier, which is still RF.

**Figure 7:**
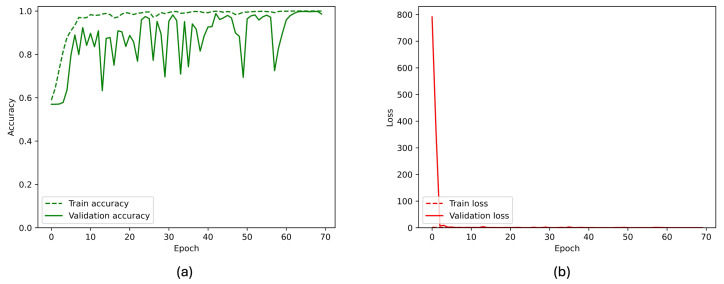
(**a**) Accuracy and (**b**) loss of the proposed InceptionResNetV2 model during the training and validation phases for the binary classification task. The x-axis represents the training epoch. Training curves are shown with a dashed line, validation curves with a solid line.

**Figure 8:**
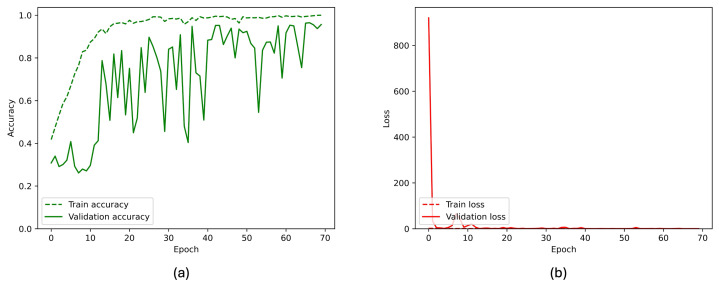
(**a**) Accuracy and (**b**) loss of the proposed InceptionResNetV2 model during the training and validation phases for the three-class classification task. The x-axis represents the training epoch. Training curves are shown with a dashed line, validation curves with a solid line.

Figure 7 and Figure 8 present the overall performance of the TL considered model, visualizing the losses and accuracies of the model in training and validation considering two classes and three classes, respectively. From these figures, it can be seen that the validation accuracy can hardly be improved fluctuating around a robust value after 70 training epochs, whereas the training accuracy steadily improves.

**Figure 9:**
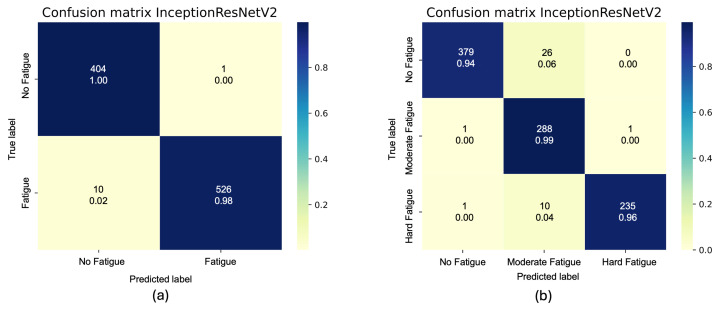
The confusion matrix on test dataset for (**a**) 2 classes and (**b**) 3 classes.

In addition, Figure 9 shows the confusion matrices of the average accuracy obtained considering two classes (a) and three classes (b). The analysis of the confusion matrices reveals a key characteristic of the model’s performance: the majority of misclassifications occur between adjacent fatigue classes (e.g., “No Fatigue” is confused with “Moderate Fatigue” and “Hard Fatigue” is confused with “Moderate Fatigue”). This pattern is highly significant, as it strongly reflects the subjective and continuous nature of muscle fatigue perception. Fatigue does not progress in discrete, abrupt jumps but rather as a smooth continuum. The model’s tendency to confuse neighboring states indicates that it has learned a representation of fatigue that captures this gradual transition, rather than making arbitrary errors. This behavior is clinically reassuring, as it suggests the system is sensitive to the progression of fatigue, making a “severely fatigued” state being misclassified as “non-fatigued” highly unlikely, which is critical for safety applications in AAL.

**Figure 10:**
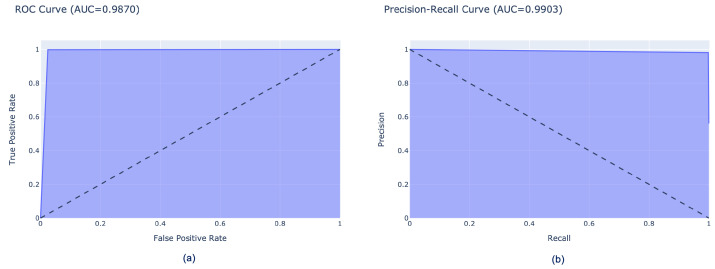
(**a**) ROC curve (AUC=0.9870) and (**b**) precision–recall curve (AUC=0.9903) of the InceptionResNetV2 model for the binary classification task. The diagonal dashed lines represent the performance of a random classifier (AUC = 0.5), serving as a baseline for comparison.

**Figure 11:**
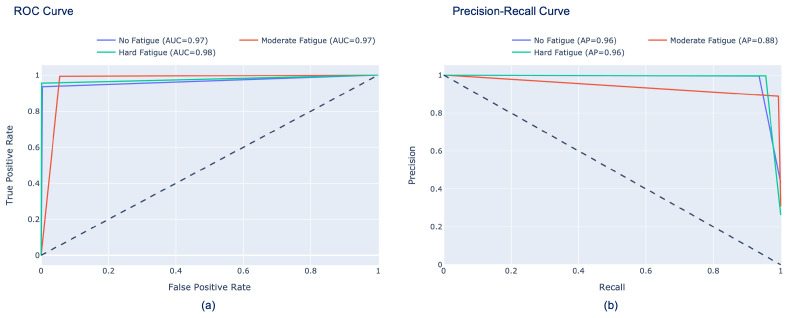
(**a**) ROC curve and (**b**) precision–recall curve of the InceptionResNetV2 model for the three-class classification task. The diagonal dashed lines represent the performance of a random classifier (AUC = 0.5), serving as a baseline for comparison.

Finally, Figure 10 and Figure 11 show the performance of our model in terms of ROC curves (a) and precision–recall curves (b), which clearly demonstrate the trade-off between the TP rate and the false positive rate for ROC curves and the trade-off between FN and FP for precision–recall curves. These curves confirm that the proposed approach is highly effective in accurately detecting muscle fatigue, achieving a score for the two classes of 98.7% AUC-ROC and 99.03% AUC–precision–recall. Similarly, the scores for the three classes are also high, as can be seen in Figure 11. In fact, the scores obtained in the AUC-ROC are 97% for No Fatigue and Moderate Fatigue and 98% for Hard Fatigue, while for AUC–precision–recall, respectively, we have 96% for No Fatigue and Hard Fatigue and 88% for Moderate Fatigue.

### 3.1. Impact of Labeling Strategy on Performance

To quantify the effect of the safety-oriented labeling bias, we performed an additional analysis comparing model performance on two distinct data subsets:

1. Full Dataset: The complete set of labeled segments, including those where the label was upgraded due to disagreement (∼12% of segments).
2. Consensus Subset: Only segments where the temporal phase and the self-reported Borg score were in agreement (∼88% of segments), representing a more neutral, albeit subjectively defined, ground truth.

The InceptionResNetV2 model was retrained and evaluated separately on each subset using the same LOSO cross-validation protocol. Specifically, the results for the three-class task, presented in Table 8, show that while performance on the full dataset remains strong, metrics on the consensus subset are marginally lower but still robust. Accuracy decreased from 95.64% to 93.2%, and mean per-class F1-score decreased from 95.91% to 92.8%. This analysis confirms that the labeling strategy inflates performance metrics, but also demonstrates that the model retains substantial discriminative power even on the less-biased consensus data. This supports the feasibility of the underlying approach while underscoring the need for objective ground-truth labels in future work.

### 3.2. Ablation Studies on Input Representation and Model

To better isolate the contributions of the input representation and the transfer learning component, we performed two ablation studies. First, a CNN trained from scratch on the CWT scalograms (identical architecture to the classification head in Figure 6, but with randomly initialized weights) achieved a mean LOSO accuracy of 89.1% (±0.025) for binary classification. This is substantially lower than the 98.6% achieved by our fine-tuned pre-trained model, indicating that transfer learning provided a significant boost beyond what the scalogram representation alone could offer to a vanilla deep model. Second, we extracted texture and shape features (Haralick features, Hu moments) from the scalograms and provided them to the best-performing classical ML classifier (RF). This “ML-on-scalogram-features” pipeline achieved 93.3% (±0.019) accuracy, which is better than ML on traditional sEMG features (92.0%) but still significantly worse than our full TL pipeline. These results suggest that while the CWT representation is more informative than traditional features, the combination of this representation with the feature extraction and generalization capabilities of a pre-trained CNN is responsible for the highest performance.

## 4. Conclusions

This study presents a novel DL-based framework for non-invasive muscle fatigue monitoring using sEMG signals, validated on a cohort of healthy adults. The combination of time–frequency representations via CWT with transfer learning on pre-trained CNNs achieves state-of-the-art performance on this dataset, demonstrating the technical feasibility of the approach.

Compared to traditional ML classifiers relying on handcrafted features, our method demonstrates higher accuracy, robustness, and generalizability across subjects. The presented comparison between our proposed pipeline and traditional ML is not a controlled ablation of the TL component alone. The superior performance stems from the synergy between the informative CWT-based time–frequency representation and the powerful, adaptable feature hierarchy of the pre-trained CNN. While our ablation studies confirm that both elements contribute significantly, future work should include a more granular comparison, such as evaluating TL against classical ML on an identical input representation (e.g., using a common set of features extracted by a pre-trained CNN). Nonetheless, the implemented end-to-end pipeline demonstrates a highly effective approach for sEMG-based fatigue assessment.

The system’s wireless hardware setup, real-time visualization capabilities, and modular classification pipeline represent a promising step toward practical fatigue detection tools for older adults.

However, a key limitation of this work is the absence of validation on clinical populations. The system’s presented utility for frail elderly individuals or those with neuromuscular diseases (e.g., sarcopenia, Parkinson’s disease) remains a hypothesis motivating future research, not a conclusion supported by the current data. Therefore, the primary contribution of this study is a robust methodological pipeline for fatigue classification in healthy individuals, which serves as an essential foundation for subsequent clinical translation. It is useful to emphasize that the present validation, utilizing a multi-electrode research-grade setup, represents a proof-of-concept in a controlled environment. The direct application of this specific hardware configuration for unsupervised daily living is not intended nor practical. The significant contribution of this work is the demonstration that a transfer learning framework applied to sEMG time–frequency representations can achieve high-accuracy, multi-level fatigue classification. This algorithmic core is the essential component that must be validated before it can be translated into viable products. The logical and necessary next step for real-world application in AAL is the integration of this validated algorithm into user-centric wearable devices. This includes designs such as sensorized garments (e.g., elbow braces used in sports) with pre-positioned, dry electrodes that simplify donning and ensure consistency, thereby moving the technology from a research tool towards a practical, low-burden aid. Therefore, this study provides the methodological foundation and performance benchmark upon which such practical implementations can be confidently developed.

Another limitation of this study is the modest sample size (N=20) for a DL approach employing complete model fine-tuning. While LOSO cross-validation provides a robust estimate of cross-subject generalizability within this cohort, future work must validate the model on larger, independent, and more diverse populations to confirm its robustness and clinical applicability. Furthermore, techniques such as federated learning or the use of larger pre-trained models from related physiological domains (e.g., trained on public sEMG datasets) could help alleviate data scarcity issues in future developments.

In addition, future work must focus on clinical validation with the intended target populations. This includes testing the framework on individuals with age-related sarcopenia, neurodegenerative disorders, and other conditions associated with elevated fatigue risk within AAL scenarios. Such studies will be necessary to assess the model’s generalizability, identify potential need for adaptation, and ultimately determine its real-world clinical efficacy and safety. In addition, other deep architectures will be compared to assess whether the choice of InceptionResnetV2 is the best in terms of overall accuracy. Furthermore, other techniques suitable for time series analysis such as LSTM will be applied in order to assess the classification accuracy of DL methods using directly acquired 1D signals.

## Figures and Tables

**Table 1:** Labeling strategy for fatigue classification.

Task Progress (%)	Self-Reported Fatigue (0–10)	Assigned Class
0–33%	0–2	No Fatigue
34–66%	3–5	Moderate Fatigue
67–100%	6–10	Hard Fatigue

**Table 2:** Mathematical formulae of the considered features for a sEMG signal segment of length K.

Features	Formulas
RMS	RMS = $\sqrt{\frac{\sum_{i=1}^{K}EMG_{i}^{2}}{K}}$
MAV	MAV = $\frac{\sum_{i=1}^{K}r_{i}\left|EMG_{i}\right|}{K}$
ZC	$\mathrm{ZC}=\sum_{i=1}^{K-1}\left[sgn\left(EMG_{i}\times EMG_{i+1}\right)\cap \left|EMG_{i+1}-EMG_{i}\right|\geq 0\right]$ $sgn\left(EMG\right)=\{\begin{array}{cc} 1, & \mathrm{if}\;EMG\geq thr\\ 0, & \mathrm{if}\;EMG<thr \end{array}$ where threshold thr = 0.1 mV
MNF	$\mathrm{MNF}=\frac{\sum_{i=1}^{K}f_{i}P\left(f_{i}\right)}{\sum_{i=1}^{K}P\left(f_{i}\right)}$ where $f_{i}$ is the frequency value of the EMG power spectrum at the frequency bin *i*, $P(f_{i})$ is the EMG power spectrum at the frequency bin *i*
MPF	$\mathrm{MPF}=\frac{\int_{0}^{\infty }fP\left(f\right)df}{\int_{0}^{\infty }P\left(f\right)df}$ *f* is sEMG frequency and P(f) is the power spectral density function of sEMG.
WL	WL = $\sum_{i=1}^{K-1}\left|EMG_{i}-EMG_{i-1}\right|$
MDF	$\mathrm{MDF}=\frac{1}{2}\sum_{i=1}^{K}P\left(f_{i}\right)$ where $P(f_{i})$ is the EMG power spectrum at the frequency bin *i*
WAMP	$\mathrm{WAMP}=\sum_{i=1}^{K}f\left(\left|EMG_{i}-EMG_{i+1}\right|\right)$ $f\left(EMG\right)=\{\begin{array}{cc} 1, & \mathrm{if}\;EMG\geq thr\\ 0, & \mathrm{if}\;EMG<thr \end{array}$ where threshold thr = 0.1 mV

**Table 3:** Hyperparameters used for the proposed TL architectures.

Hyperparameter	Values
Learning rate	0.002
Batch size	128
Optimizer	Adam
Output activation	softmax
number of epochs	70

**Table 4:** Parameters used for ML models with two classes.

Model	Parameters
LR	solver = newton-cg, max_iter = 60, multi_class = ovr, C = 0.002
RF	max_depth = 15, n_estimators = 12, criterion = gini
DT	criterion = gini, max_depth = 13
SVM	decision_function_shape = ovo, max_iter = 80, kernel = linear, C = 0.3
KNN	n_neighbors = 7, metric = minkowski, algorithms = auto, weights = distance

**Table 5:** Parameters used for ML models with three classes.

Model	Parameters
LR	solver = newton-cg, max_iter = 65, multi_class = ovr, C = 0.003
RF	max_depth = 14, n_estimators = 14, criterion = gini
DT	criterion = gini, max_depth = 16
SVM	decision_function_shape = ovo, max_iter = 75, kernel = linear, C = 0.4
KNN	n_neighbors = 8, metric = minkowski, algorithms = auto, weights = distance

**Table 6:** Mean and standard deviation classifier results with considered metrics for two classes. The best-performing results are highlighted in bold.

Model	Accuracy	Precision	Recall	F1-Score
InceptionResNetV2	**0.9862 (±0.012)**	**0.9885 (±0.011)**	**0.9894 (±0.013)**	**0.9883 (±0.011)**
DenseNet121	0.9490 (±0.01)	0.9492 (±0.013)	0.9489 (±0.008)	0.9490 (±0.006)
ResNet50	0.9766 (±0.013)	0.9756 (±0.01)	0.9755 (±0.014)	0.9756 (±0.009)
InceptionV3	0.9766 (±0.011)	0.9766 (±0.008)	0.9762 (±0.011)	0.9766 (±0.005)
LR	0.7689 (±0.023)	0.7678 (±0.017)	0.7572 (±0.013)	0.7670 (±0.021)
RF	0.9201 (±0.015)	0.9200 (±0.018)	0.9162 (±0.021)	0.9200 (±0.012)
DT	0.8679 (±0.031)	0.8682 (±0.026)	0.8641 (±0.021)	0.8680 (±0.015)
SVM	0.7806 (±0.014)	0.7798 (±0.023)	0.7586 (±0.011)	0.7766 (±0.013)
KNN	0.8786 (±0.014)	0.8781 (±0.023)	0.8697 (±0.031)	0.8782 (±0.027)

**Table 7:** Mean and standard deviation classifier results with considered metrics for three classes. The best-performing results are highlighted in bold.

Model	Accuracy	Precision	Recall	F1-Score
InceptionResNetV2	**0.9564 (±0.013)**	**0.9623 (±0.012)**	**0.9614 (±0.017)**	**0.9591 (±0.011)**
DenseNet121	0.7683 (±0.013)	0.8316 (±0.015)	0.7671 (±0.006)	0.7708 (±0.003)
ResNet50	0.8363 (±0.014)	0.8863 (±0.010)	0.8136 (±0.017)	0.8350 (±0.016)
InceptionV3	0.9113 (±0.01)	0.924 (±0.016)	0.9056 (±0.019)	0.9108 (±0.009)
LR	0.6198 (±0.022)	0.6146 (±0.031)	0.5916 (±0.024)	0.6101 (±0.028)
RF	0.8637 (±0.015)	0.8626 (±0.014)	0.8518 (±0.017)	0.8617 (±0.021)
DT	0.7657 (±0.032)	0.7655 (±0.028)	0.7594 (±0.024)	0.7654 (±0.026)
SVM	0.6964 (±0.022)	0.6995 (±0.021)	0.6534 (±0.025)	0.6769 (±0.012)
KNN	0.7732 (±0.031)	0.7762 (±0.029)	0.7584 (±0.028)	0.7719 (±0.027)

**Table 8:** Full dataset vs. consensus subset mean and standard deviation classifier results with considered metrics for InceptionResNetV2 model considering 3 classes. The best-performing results are highlighted in bold.

Model	Accuracy	Precision	Recall	F1-Score
Full Dataset	**0.9564 (±0.013)**	**0.9623 (±0.012)**	**0.9614 (±0.017)**	**0.9591 (±0.011)**
Consensus Subset	0.932 (±0.012)	0.9411 (±0.012)	0.9392 (±0.005)	0.928 (±0.001)

## Data Availability

The data presented in this study are available on request from the corresponding author. The data are not publicly available due to restrictions (their containing information that could compromise the privacy of research participants).
